# The Challenges of Developing a Participatory Arts Intervention for Caregivers of Persons with Dementia

**DOI:** 10.7759/cureus.1154

**Published:** 2017-04-11

**Authors:** Adam Golden, Denise Gammonley, Gay Hanna Powell, Thomas T Wan

**Affiliations:** 1 Internal Medicine, University of Central Florida College of Medicine; Orlando VAMC; 2 College of Health and Public Affairs, University of Central Florida; 3 Ex-Director, National Center for Creative Aging

**Keywords:** dementia, caregiving, humanities, creative arts

## Abstract

Objective: We describe the development and challenges in implementing a web-based participatory art intervention specifically designed for caregivers of persons with dementia to use at home with their loved one.

Method: An interprofessional team, including an experienced national panel of artists, developed a participatory arts toolkit consisting of seven web-based modules involving a combination of music, singing, dancing, poetry, and painting. Participants completed a survey of demographics, caregiver needs, and caregiver burden.

Results: Thirty caregivers with high caregiver needs and a high caregiver burden volunteered to pilot the intervention. Difficulties with caregiver recruitment and compliance with lesson plans were noted. Caregivers provided positive and negative qualitative feedback.

Discussion: The challenges and possible solutions to the problems identified in the implementation and assessment of this participatory arts intervention will provide important insights for future studies linking the arts and dementia care.

## Introduction

There are currently no medical interventions or pharmacological therapies that will delay or cure Alzheimer’s disease [1-2]. Caregivers and health care professionals must, therefore, rely on non-pharmacological approaches in the chronic management of dementia [3]. The need to provide continuous supervision and the perceived lack of appreciation makes the caring for a family member with dementia more stressful than caring for a loved one with a disability [4]. The long-term caregiving burden causes financial as well as psychophysiological problems for the caregivers [5].  Almost one-third of dementia caregivers suffer from depression [6].  

Studies involving non-pharmacological treatments for Alzheimer’s disease have included art therapy, music therapy, dance or movement therapy, and reminiscence therapy. A review by Noice, et al. discussed the reported clinical and psychological benefits of participatory arts interventions involving dance, expressive writing, music, theater, and visual arts [7].  

Many art intervention studies involve non-controlled trials or anecdotal reports of small numbers of participants. A systematic review found no significant impact for aromatherapy, light therapy, therapeutic touch, and individualized activity on agitation in nursing home residents with dementia [8]. Similarly, a review of exercise programs showed no impact on addressing behavioral abnormalities or depression in patients with dementia [9]. A recent randomized study comparing music therapy, listening to music, and standard care in persons with dementia in nine Italian nursing homes found no differences in behavior symptoms [10]. Difficulty demonstrating a decrease in dementia-related behavioral abnormalities may be due to an underlying selection bias, as “difficult” patients are less likely to engage in an interactive sensory arts-based intervention. 

The essential components of art interventions in the real world setting are difficult to identify since best practices for therapeutic participatory sensory art interventions currently do not exist [11]. There is no guidance for health care professionals as to which specific creative arts interventions might be most appropriate for an individual patient or caregiver. The ideal “dosage” of arts-based interventions remains unknown as well.  

Many of the sensory art-based studies are conducted in long-term care facilities. Little is understood in relation to the feasibility of delivering such activities in home- and community-based settings where persons with Alzheimer’s and related dementias often receive care. No systematically constructed experimentation has been executed to compare how different sensory arts modalities may induce the reduction of specific symptoms related to caregiving burden. This article describes the development and implementation of a free web-based sensory arts “toolkit” and the challenges measuring its impact on caregiver burden.

## Materials and methods

### Development of the online artistic “toolkit”

A four-day summit of experts in gerontology and geriatric medicine, along with national experts in dementia-related art interventions from around the nation, was hosted by the University of Central Florida. Each of the participating artists had experience creating an arts program for persons with dementia (Table 1).

**Table 1:** Collaborating Artists for the Development of a Web-based “Artistic Toolkit”

Artist	Program	Link
Gary Glazner	Alzheimer’s Poetry Project, Brooklyn, NY	www.alzpoetry.com
Anne Basting	Timeslips, Milwaukee, WI	www.timeslips.org
Maria Genne	Kairos Dance, Minneapolis, MI	www.kairosalive.org
Judith Friedman	Songwriting Works, San Francisco, CA	www.songwritingworks.org
Elizabeth Lokon	Opening Minds, Miami, OH	http://miamioh.edu/cas/academics/centers/scripps/community-connections/opening-minds-through-art/index.html

From these summits and reviews of the medical and gray literature, the team decided to develop an “artistic toolkit” that would allow the caregiver to choose from among a variety of participatory art interventions. The term “participatory arts” involves an active role in the art intervention rather than a more passive role as an observer [7]. The goal of the toolkit would be to provide support for caregiver stress, loneliness, and sense of well-being through a program that allows for participation in creative-based activities with their loved one [12]. Three follow-up face-to-face meetings were conducted to discuss the content of the toolkit and the plans for its implementation and evaluation. 

Seven specific creative sensory activity lessons were developed in an online platform through the National Center for Creative Aging that involve one or more modalities, including poetry, music appreciation, singing, dancing, and the creation of paintings and collages [13]. A master artist facilitates each session to demonstrate the activity and coach the dementia patient-caregiver dyad. Lessons range in length from 8 to 20 minutes with the opportunity to easily view segments of lessons or to view an entire lesson at once. All lessons start with controlled breathing and warming exercises.

To further support learners in acquiring the skills needed to effectively use the tutorials, the caregiver toolkit includes a welcome video and a “basics” video that together provide an overview of instructions on how to use the site. There is also a short tutorial devoted to caring for the caregiver. Careful attention was paid to the usability of the website by older adults [14-15]. Usability of the website was tested by an interprofessional gerontological research team and by caregivers of adult day care center clients with dementia.

### Enrollment

Caregivers of patients with dementia were voluntarily enrolled through referrals from a community-based agency that provides adult day care services to older adults across Central Florida. Enrollment occurred during between April 15, 2015 and September 15, 2015. Case management staff provided an in-person orientation to the caregiver regarding the use of the online platform. Caregivers were offered gift certificates at a local supermarket at enrollment and at the completion of the study. Caregivers were given a link and password to the online artistic toolkit activities. Case managers called participants on a weekly to bi-weekly basis in order to encourage and track progress through the modules. Exclusion criteria involved caregivers who a) refused to sign an informed consent; b) did not have computer access; c) did not speak English; or d) care for persons with dementia who were receiving physical, occupational, or speech therapy.

### Assessment measurements 

Patient demographics (age, gender, race, ethnicity, marital status) were obtained by the community agency staff in the first assessment. Each client had an assessment of their need to assist their loved one with activities of daily living. Case management also collected caregiver demographics, relationship to the client, caregiver work status, and the number of hours of direct caregiving per week. A 12-item short version of the Zarit Burden Interview [16] was used to assess caregiver burden. The response options for each question item range from 0 (never) to 4 (nearly always); higher scores indicate a greater level of caregiving burden.  

One month after enrollment, caregivers were contacted through an in-person or telephone-based interview by a care manager at the adult day care center. Qualitative data regarding satisfaction with the toolkit, adherence and barrier issues, frequency, and intensity of specific artistic interventions was also obtained by the adult day care center staff. 

## Results

Forty-two clients with dementia were identified. Thirty-two clients consented to participate in the pilot of this intervention. Thirty caregivers completed all the initial surveys modules and surveys after multiple attempts at prompting from the adult day care center case managers. Three caregivers asked to be withdrawn from the study after completing the initial survey due to reluctance to complete the online lessons and follow-up surveys. Demographics of the adult day care clients with dementia are presented in Table 2.  

**Table 2:** Demographics of Adult Day Care Clients with Dementia (N=27) SD: standard deviation

Years of age (average, ± SD)	79.8 ( ± 11.4)
Females	18 (66.7%)
Race/Ethnicity	
White	8 (29.6%)
Black	9 (33.3%)
Hispanic	10 (37.0%)
Need assistance with one or more activities of daily living	17 (63.0%)

The distributions of important caregiver demographic variables are presented in Table 3.

**Table 3:** Baseline Demographics of Dementia Caregivers (N=27) SD: standard deviation

Demographics	
Years of age (average, ± SD)	53.3 (± 13.1)
Female	26 (96.3%)
Race/Ethnicity	
White	10 (37.0%)
Black	8 (29.6%)
Hispanic	8 (29.6%)
Other	1 (3.7%)
Married	24 (88.9%)
Relationship between caregiver and care recipient	
Daughter	17 (63.0%)
Spouse	7 (25.9%)
Other	3 (11.1%)
Employed outside home	14 (51.9%)

Although nine caregivers identified themselves as Hispanic, all of the clients had an English-speaking caregiver. Of the 27 caregivers included in the analysis, the mean age was 54 (SD = ± 11.8). Daughters constituted 63% of clients’ caregivers. The baseline mean Zarit score was 34.6 ± 7.0 (normally less than 17) [16]. All caregivers reported spending more than 40 hours per week caring for their loved one with dementia.

Only 11 caregivers completed all seven modules. During the analysis period, there were several prolonged periods where the server for the online lessons was not working. Qualitative feedback revealed that the caregivers enjoyed being able to interact with their family member with dementia at home and spend quality companion time rather than caregiving time. The controlled breathing and warming exercises were well appreciated. Several specific comments from the caregivers include:

“Introduced strategies that they would never have thought of at home.”

“We really enjoyed the activities because it allowed us to do more than just sit around.”

Caregivers noted that the lessons do not take into account the large functional and cognitive variability among dementia patients. The most common complaint by caregivers was gathering the supplies needed to complete the lesson. There was a concern that some of the lessons could be financially costly if caregivers do not already have the supplies at home. Several caregivers recommended providing alternatives or substitutions for supplies that may already be in the home. Interestingly, one caregiver reported an underlying poor long-term relationship with the client that made the interaction of the lesson plans awkward and non-beneficial. No caregivers reported any adverse events associated with the use of the online lesson plans.

## Discussion

### Conceptual model building

This study describes the development and challenges implementing an online platform for a participatory sensory arts intervention for dementia caregivers experiencing a high caregiver burden. A number of key innovations differentiate this effort from other efforts to develop art-based interventions. The seven lesson plans offer a wide variety of artistic intervention options to address the heterogeneity of patient-caregiver life experiences, personal preferences, patient cognitive impairment, and levels of functional impairment. In this intervention, the caregiver is an active participant in the activities and the primary focus of assessment. Given that less than one-fourth of the caregivers were a spouse, the web-based format also offers the opportunity for intergenerational engagement.

The utilization of a web-based platform allows accessibility across geographic areas to be utilized by homebound older adults. Nursing homes are a natural setting to test the effectiveness of a non-pharmacological intervention given the availability of subjects and personal health information. However, to test the impact on caregivers, a home-based setting is preferable but difficult to conduct due to the relative social and geographic isolation of dementia patient-caregiver dyads [17]. 

### Practical challenges

The population agreeing to participate in this pilot needed much prompting and follow-up by the care managers. Even with an intervention with no side-effects and a monetary incentive, caregiver enrollment and compliance were still a challenge. We suspect that there was much reluctance among caregivers to “add” a new activity to their already busy and burdensome days.

Completion of all seven lessons and completion of the post-intervention survey was low but not surprising given the presumed heterogeneity in artistic interest among persons with dementia and their caregivers. Utilization of caregiver support groups might offer a different avenue to recruit caregivers who might be more eager to engage in new nonpharmacologic interventions. 

As a result, we were unable to measure a “therapeutic” benefit from the intervention. A direct measurement of the artistic dosage of use also did not occur. Similarly, we did not assess compliance with the lesson plans. Because the online lesson plans were designed to be easy to implement, caregivers should be able to adopt the use of this intervention “offline.” Website usage alone does not necessarily correlate with intensity of artistic intervention. Measuring impact on patient healthcare outcomes, as well as caregiver well-being is both complex and resource intensive. 

## Conclusions

Based on our experience, future studies will need to focus on developing trials that measure the correlation between a “dose response” and potential caregiver benefits. Such studies will require measuring participatory arts usage and caregiver survey data to assess the frequency and intensity of sensory art intervention use. Related to this question is developing an understanding of the factors that motivate caregivers to choose a specific arts-based modality. These efforts will provide healthcare professionals and caregivers with the information needed to develop a “sensory arts prescription” for patients with dementia and their caregivers.
